# Study and Characterization of Polyvinyl Alcohol-Based Formulations for 3D Printlets Obtained via Fused Deposition Modeling

**DOI:** 10.3390/pharmaceutics15071867

**Published:** 2023-07-02

**Authors:** Sofiya Ilieva, Dilyana Georgieva, Valentina Petkova, Milen Dimitrov

**Affiliations:** 1 Department of Pharmaceutical Technology and Biopharmacy, Faculty of Pharmacy, Medical University of Sofia, 2 Dunav Str., 1000 Sofia, Bulgaria; sofiailieva91@gmail.com (S.I.); dgeorgieva@pharmfac.mu-sofia.bg (D.G.); 2 Department of Organisation and Economics of Pharmacy, Faculty of Pharmacy, Medical University of Sofia, 2 Dunav Str., 1000 Sofia, Bulgaria; vpetkova@pharmfac.mu-sofia.bg

**Keywords:** three-dimensional printing, fused deposition modeling, polyvinyl alcohol, hot melt extrusion, Pareto chart, quality by design (QbD)

## Abstract

Three-dimensional (3D) printing has emerged as a new promising technique for the production of personalized dosage forms and medical devices. Polyvinyl alcohol is prominently used as a source material to produce 3D-printed medicines via fused deposition modeling (FDM)—a technology that combines hot melt extrusion and 3D printing. A preliminary screening of three grades of PVA indicated that partially hydrolyzed PVA with a molecular weight (MW) of 31,000–50,000 and plasticized with sorbitol was most suitable for 3D printing. Paracetamol was used as a model drug. The materials and the produced filaments were characterized by X-ray powder diffraction (XRPD), thermogravimetric analysis (TGA), and differential scanning calorimetry (DSC). The complex viscosity (η*) of the polymer melts was determined as a function of the angular frequency (ω) at the printing temperature to assess their printability. Three-dimensional printlets with a 40% infill exhibited an immediate release of the API, while tablets with a higher infill were prone to a prolonged release regardless of the filament drug loading. A factorial design was used to give more insight into the influence of the drug-loading of the filaments and the tablet infill as independent variables on the production of 3D printlets. The Pareto chart confirmed that the infill had a statistically significant effect on the dissolution rate after 45 min, which was chosen as the response variable.

## 1. Introduction

Three-dimensional (3D) printing has emerged as a new promising technique for the production of personalized dosage forms and medical devices. It has many advantages over conventional pharmaceutical technologies, including personalized dosing for specific groups of patients, combining multiple drugs with varying concentrations in individual “polypills”, and customization of the drug release. The small size and the ability of 3D printers to connect with a healthcare database allow for the production of small batches or even individual medicines for every patient at the point of care (hospital or community pharmacy) [1,2,3]. As 3D printing creates objects with versatile and complex geometries, it allows for the production of implants [4], patient-specific medical devices [5,6], and unconventional tablet shapes [7,8]. The principal mechanisms of 3D printing are a powder bed, photopolymerization, selective laser sintering, and extrusion-based methods, the latter being semi-solid extrusion and fused deposition modeling (FDM) [1,3,9]. The release kinetics of FDM 3D-printed dosage forms is widely reported to depend on the properties of the used polymer, the geometry of the printed object, and its infill percentage. The drug loading of the polymer matrix could have an impact on the drug release [2,10,11].

Concerning FDM for pharmaceutical applications, the incorporation of the active pharmaceutical ingredient into the filament is crucial. The two principal methods reported in the literature are soaking a commercial filament in a solution of the active pharmaceutical ingredient (API) [12] and hot melt extrusion (HME) [5,7,8,13,14]. The drug loading achieved by soaking is low, and thus, it is suitable for low-dose preparations. In addition, the substance should be soluble and the filament insoluble in the particular solvent [12]. Hot melt extrusion allows for the inclusion of a higher percentage of active ingredient(s) [13] and has the advantage of increasing the solubility and bioavailability of poorly water-soluble drugs [1].

Different thermoplastic pharma-grade polymers, such as polyvinyl alcohol [14], hypromellose [15], hydroxypropyl cellulose [15], ethyl cellulose [15,16], and methacrylic acid copolymers [13,17] have been screened for the production of FDM filaments. Partially hydrolyzed polyvinyl alcohol (PVA) was chosen for this study. It is a hydrophilic, non-toxic, semi-crystalline water-soluble polymer that enhances bioavailability by forming solid amorphous dispersions [18,19], which makes it suitable for the production of solid oral dosage forms. It is also a source material for commercial filaments [7,12]. However, it is seldom used in HME because of its high processing temperature and the narrow interval between its glass transition (Tg) and degradation temperature (Tdeg). There have been successful attempts to extrude partially hydrolyzed grades by adding hydrophilic plasticizers containing hydroxyl groups, such as glycerin [20] and sorbitol [19], which form hydrogen bonds with the PVA molecule and lower its Tg. Such experiments have also been conducted focusing on the production of strands for FDM 3D printing for pharmaceutical applications [14,21,22,23,24].

The design of experiments (DoE) and quality by design (QbD) principles have recently been implemented in studies involving FDM 3D printing [11,14,22,25,26,27,28]. QbD uses both science and quality risk management in order to obtain thorough knowledge about the products, processes, and process control [29]. Some researchers have applied DoE to filament extrusion, analyzing the effect of the process parameters on filament quality [26]. Others have concentrated entirely on the filament formulation, applying a mixture design to evaluate the combined effect of different excipients on the drug release of the 3D-printed tablets [27]. However, more experimental designs gravitate towards the printing parameters [14,22,28] or a combination of the printing parameters and filament formulation [11,25]. The defined response variables usually involve drug release and occasionally solvent extraction [11], tablet mass, and manufacturing time [28]. The interpretation of the results has generally included ANOVA and response surface methodology (RSM) [14,25,27,28]. Although the Pareto chart is a widely used statistical tool that evaluates the effect of the input variables on the defined responses [30,31], it has only been reported once in relation to the 3D printing of medicines [25].

The aim of the present study was to evaluate the influence of two input variables (the drug loading of extrudates and the infill percentage of tablets) on the dissolution rate of 3D printlets using statistical approaches. Three grades of partially hydrolyzed PVA were tested as potential excipients for FDM pharmaceutical dosage forms. To the authors’ best knowledge, this is the first systematic screening of the suitability of different grades of PVA for the production of filaments for 3D printing for pharmaceutical applications.

## 2. Materials and Methods

### 2.1. Materials

Three grades of polyvinyl alcohol with varying molecular weight (MW) were used in this study. “PVA 1”, with a MW of 13,000–23,000 and 87–89% degree of hydrolysis, and “PVA 2” with a MW of 31,000–50,000 and 87–89% degree of hydrolysis, were purchased from Acros Organics (Geel, Belgium). “PVA 3”, with a MW of 70,000 and 87–89% degree of hydrolysis, was acquired from Valerus (Sofia, Bulgaria). Polyethylene glycol 6000 “PEG” (Clariant Produkte, Wiesbaden, Germany) and sorbitol “Sorb” (DHW, Dessau-Roßlau, Germany) were used as plasticizers. Magnesium stearate, “MgST”, was purchased from Union Derivan (Barcelona, Spain). Paracetamol (Hebei Jiheng Pharmaceutical Co., LTD, Hengshui, Hebei, China) was used as a model drug. Commercial PVA filament was purchased from Shenzhen Lankeda Technology Co., Ltd. (Shenzhen, China).

### 2.2. Methods

#### 2.2.1. Extrusion and 3D Printing

- Extrusion of the placebo filaments

The placebo formulations were labeled from E1 to E12. In the first stage of the survey (Table 1), the above-mentioned three grades of PVA, combined with either PEG or sorbitol, were screened for their suitability for 3D printing. The polymers and the plasticizers were accurately weighed and mixed in ratios 10:1.11, and the samples of approximately 100 g were processed through a 16 mm single screw extruder (Wellzoom, Shenzhen, China) with two heating zones and 1.80 mm die to produce extrudates with 1.75 mm diameter. Bearing in mind the limited capacity of the single-screw filament extruders, the excipients were granulated to ensure the homogeneity of the extrudates. The process was different depending on the plasticizer. PEG 6000 was melted and added to PVA in a Beaker glass; the mixture was homogenized with a spatula and calibrated through a 3.0 mm sieve. The produced granules were left to cool at room temperature. For the samples with sorbitol, a 50% w/w water solution of sorbitol was added to the PVA in a beaker glass; the mixture was homogenized with a spatula, calibrated through a 3.0 mm sieve, and dried in a tray dryer for 3 h at 80 °C. The produced granules were extruded at 10 rpm with the process temperature adapted depending on the MW of the polymer (185 °C for PVA 1, 200 °C for PVA 2, and 215 °C for PVA 3).

In the second stage of the study, the composition (E4) was optimized by increasing the amount of the plasticizer (Table 1). Each variation of the sorbitol quantity was processed with and without magnesium stearate in order to explore the effect of the lubricant upon the extrusion. Filament E10 (labeled “fixed mixture of PVA 2 and sorbitol” or PVAS) was chosen as a basic ingredient for the upcoming extrusion of the drug-loaded filaments.

- Extrusion of the drug-loaded filaments

The drug-loaded filaments P5, P10, and P15, containing PVAS and 5%, 10%, and 15% paracetamol, respectively, were prepared like the placebo filaments. The API and the polymer were homogenized in a beaker glass, the sorbitol solution was added, and the mixture was additionally homogenized. After calibration through a 3.0 mm sieve, the granules were dried in a tray dryer for 3 h at 80 °C. The granulated sample was processed through the extruder at 200 °C and with a screw speed set at 10 rpm to produce 1.75 mm filaments.

- 3D printing of the placebo object

All objects were designed in AutoCAD, exported in a .stl file, and loaded into the Simplify 3D software prior to printing. A simple geometry shape (a disc with d = 35 mm and h = 1 mm) was chosen to be printed from the placebo strands to test the basic printability as a critical quality attribute (CQA) of the obtained in-process filaments. The discs were printed with a 3D printer Delta Rostock mini G2S pro (Shenzhen, China) with a 0.4 mm die and 100% infill percentage. The printing temperature was set at 185 °C. The 1 mm height structure was built by applying five consecutive 0.2 mm polymer layers.

- 3D printing of the tablets

The 3D-printed tablets called printlets [10] were produced using the above-mentioned printer. The oval-shaped tablets (14 mm/7 mm/4 mm) were printed with a 0.4 mm nozzle diameter, 0.2 mm layer height, 185 °C extrusion temperature, a rectilinear internal fill pattern with 40, 70, or 100% infill depending on the experiment, and a concentric external fill pattern. The first layer was printed with 150% width and 20% speed to provide better adhesion to the building plate. 

#### 2.2.2. Morphology

The diameter of the produced strands was monitored with a digital caliper. A light microscope (Marcel Aubert, Nidau, Switzerland) with a VideoPIC application was used to obtain images of the tablets. Additionally, the surface of a tablet obtained from experiment P5E40 was examined with a scanning electron microscope (SEM) Tabletop SEM HIROX SH-4000M (HIROX Europe, Limonest, France). The sample was attached to a SEM stub using a double adhesive tape and then sputtered with gold. The images were captured using an electron beam with an accelerating voltage of 15 kV, with 100× magnification.

#### 2.2.3. X-ray Powder Diffraction (XRPD)

The physical form of the materials was assessed by X-ray powder diffraction. The XRPD patterns were obtained in a Bruker D8 Advance using Cu Kα X-ray source and LynxEye position-sensitive detector. The intensity and the voltage applied were 40 mA and 40 kV. The angular range of the data acquisition was 5.5–80° 2θ with a step of 0.02° 2θ and 10 s/step counting statistics. 

#### 2.2.4. Assay of the Extruded Filaments

Prior to printing, the amount of paracetamol in the extrudates was analyzed via UV/VIS spectrophotometer (RayLeigh UV9200, Beijing, China). Approximately 100 mg pieces of extrudate from each formulation were weighed, transferred into a volumetric flask, and made up to 100 mL with phosphate buffer (pH 5.80). One mL of this solution was diluted up to 20 mL in another volumetric flask. The absorption was measured at 243 nm, and the concentration of the API was calculated using a standard curve method. Each measurement was done in triplicate. The drug-loading percentage was obtained by dividing the amount of the API in each piece of extrudate by the mass of the piece of extrudate.

#### 2.2.5. Thermogravimetric Analysis

The thermogravimetric analysis (TGA) was performed with a TGA4000 Thermo-gravimetric Analyzer (PerkinElmer, Waltham, MA, USA) to measure the water content and the degradation temperature of the raw materials (PVA, sorbitol, and paracetamol), the physical mixture, the granules and the filaments, containing PVA, sorbitol, and paracetamol. The samples (about 20 mg) were accurately weighed and placed in an aluminum pan. The samples were heated from 40 °C to 800 °C at a heating rate of 10 °C/min under 20 mL/min argon gas for purging. The data were recorded and analyzed with Pyris TM software (PerkinElmer, Waltham, MA, USA).

#### 2.2.6. Differential Scanning Calorimetry

Differential scanning calorimetry (DSC) 4000 system (PerkinElmer, Waltham, MA, USA) was used to analyze the thermal properties of the raw materials (PVA, sorbitol, and paracetamol), the physical mixture, and the filaments containing PVA, sorbitol, and paracetamol. The samples (approximately 10 mg) were accurately weighed and placed in an aluminum pan. The experiments were conducted under an argon environment (flow rate 20 mL/min). The samples were cooled up to −70 °C with an IntraCooler III cooling accessory and heated from −70 °C to 200 °C at a heating rate of 10 °C/min. The data were processed with Pyris TM software (PerkinElmer, Waltham, MA, USA).

#### 2.2.7. Rheology

The complex viscosity (η*) of the formulations E8, E10, E12, P5, P10, and P15 and the commercial PVA filament was determined as a function of the angular frequency (ω). The rheological analysis was conducted using a rotational rheometer AR-G2, TA Instruments, New Castle, DE, USA. The electrical heating parallel plate geometry with crosshatched plates of 25 mm diameter at a fixed gap of 1 mm between the plates was used. Prior to testing, the material was heated up to 170 °C and pressed into a 22 mm slug using Carver hydraulic press (Carver Inc., Wabash, IN, USA). Preliminary strain sweep tests were carried out for all samples in the strain (γ) range of 0.01–100% at a fixed frequency of 1 Hz to determine the linear viscoelastic region (LVR) and to choose the strain amplitude for the consecutive frequency sweep measurements. An oscillation frequency sweep study was conducted by varying the angular frequency from 0.1 to 100 rad/s at a fixed temperature of 185 °C in order to measure the complex viscosity. The complex viscosity of the commercial PVA filament with proven printability was used as a reference.

#### 2.2.8. In Vitro Drug Release

The dissolution test of the tablets was performed according to Ph. Eur. 2.9.3 apparatus II (paddle) (Copley Dis 8000, Nottingham, UK) in phosphate buffer (pH 5.80); temperature 37 ± 0.5 °C, volume 500 mL; stirring speed 50 rpm. A total of 5 mL samples were taken after 5, 15, 30, 45, 60, 90, 120, 240, 300, and 360 min with media replacement. The quantity of the released paracetamol from the 3D-printed tablets was measured spectrophotometrically. The absorption was measured at 243 nm, and the API content was calculated using the standard curve method described in Section 2.2.4. All measurements were done in triplicate.

#### 2.2.9. Drug Release Kinetics

Four kinetic models, such as Zero-order, First-order, Higuchi, and Korsmeyer–Peppas were applied in order to study the drug release process from the different models.

- Zero-order model—this model refers to drug release processes whose rate does not depend on the drug concentration.

(1) $$\frac{M(t)}{M(\infty )}=k_{0}t$$

- First-order model—this model describes the release process when the drug release rate is proportional to the drug concentration, i.e., a constant fraction of the drug is released per unit of time.

(2) $$\frac{M(t)}{M(\infty )}=e^{-k_{1}t}$$

- Higuchi model—this model assumes that two mechanisms are responsible for controlling the drug release rate: swelling and erosion/degradation. In the Higuchi model, k_H_ is a constant proportional to the burst release rate of the release process.

(3) $$\frac{M(t)}{M(\infty )}=k_{H}t^{\frac{1}{2}}$$

- Korsmeyer–Peppas model—this model is used to describe the drug release process in occasions when the release follows several kinetics mechanisms.

(4) $$\frac{M(t)}{M(\infty )}=k_{KP}t^{n}$$

In the equations, M(t) represents the amount of paracetamol released at time t, and M(∞) represents the total amount of paracetamol included in the tablets; k_0_, k_1_, k_H,_ and k_KP_ are the constants of the Zero-order, the First-order, the Higuchi and the Korsmeyer–Peppas models.

#### 2.2.10. Design of the Experiments

A 2^2^ full factorial design with two repetitions of the center point was used for the development of the 3D-printed tablets. Two continuous factors (the drug loading of the filaments and the infill percentage of the tablets) were chosen as input variables. The percentage of the released drug after 45 min was defined as a response variable. The prepared filaments with different drug loading, P5, P10, and P15, were used as a source material for printlets with 40%, 70%, and 100% infill. The experimental runs were coded P5I40, P15I40, P5I100, P15I100, P10I70-1, and P10I70-2 (see Table 2). The statistical evaluation was performed using Minitab software (ver. 16.0; Minitab Inc, State College, PA, USA). The effect of the independent variables on the response was demonstrated by a Pareto chart and a Contour Plot.

## 3. Results and Discussion

### 3.1. Extrudability and Printability

In the first stage, the extrudates E1 and E2 were too brittle after cooling and could not be loaded in the feeding gear of the printer. They also exhibited a rough surface and an uneven diameter. The filaments E3 to E6 had adequate mechanical properties to be successfully loaded into the 3D printer. However, surface roughness was also observed for the PEG-containing formulations (E3 and E5). This phenomenon could be explained by the properties of polyethylene glycol and the construction of the particular extruder. The first heating zone was situated too close to the hopper and caused softening of PEG due to its low melting temperature. As a result, the plasticizer was not conveyed steadily through the barrel, and the homogeneity of the extrudate was compromised, which resulted in “shark skin” appearance and inconsistent diameter of the filament. The filaments E5 and E6 had sufficient flexibility to be loaded into the feeding gear of the 3D printer but swelled inside the nozzle and blocked it, probably because of the higher melt viscosity of PVA with a MW 70,000. The filaments E3 and E4 successfully passed through the nozzle and were printed into a predesigned structure. The PEG-containing filament E3 was characterized by roughness and an inconsistent diameter leading to inaccurate feeding of the printer and poor quality of the produced object. It was established that E4, containing PVA 2 combined with sorbitol, was most promising for 3D printing, although it lacked enough flexibility. A disc with a predefined shape was successfully printed from the sorbitol-containing E4 strand, indicating that it fulfilled the CQA of an intermediate for 3D printing—“printability”. However, the filament had a slight tendency to break while loading due to insufficient plasticity. The printability of the formulations from stages 2 and 3 was further investigated by comparing their complex viscosity curves at printing temperature, as discussed below.

**Figure 1:**
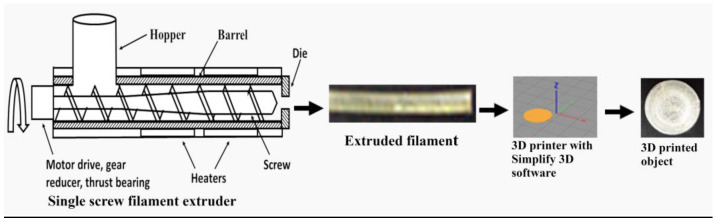
Flow chart of extrusion and 3D printing process.

Sorbitol proved to be a more efficient plasticizer, and the promising formulation E4 was optimized in the second stage of the survey by increasing its quantity (see Table 1). Each composition was prepared with and without magnesium stearate in order to assess if the inclusion of a lubricant would result in decreasing the friction during extrusion and subsequently lowering the process temperature. The compositions E7 to E10 were successfully extruded into filaments with favorable mechanical properties and were printed into discs, while E11 and E12 exhibited an uneven diameter, possibly due to overplastification. The inclusion of magnesium stearate had no influence on the process; thus, the lubricant was found to be unnecessary for the formulation. It could be concluded that PVA with a MW 31,000–50,000 could be extruded into filaments with or without a lubricant when the polymer:sorbitol ratio is between 10:1.25 and 10:1.50, which corresponds to 11% to 13% of sorbitol. A flow chart of the process is represented in Figure 1.

In the third stage, the drug-loaded filaments containing 5%, 10%, and 15% paracetamol and 95%, 90%, and 85% PVAS, respectively, were successfully extruded and printed into tablets. They contained, respectively, 12.4%, 11,7%, and 11.1% plasticizer, which was in accordance with the stated optimal quantity of the plasticizer between 11% and 13%. Batches of 10 tablets were printed from each drug-loaded filament as outlined in Table 2. The printing time for the different tablets depended on their infill percentage: approximately 2 min per tablet for P5I10 and P15I40, 3 min for P10I70-1 and P10I70-2, and 4 min for P5I100 and P15I100.

### 3.2. Morphology

**Figure 2:**
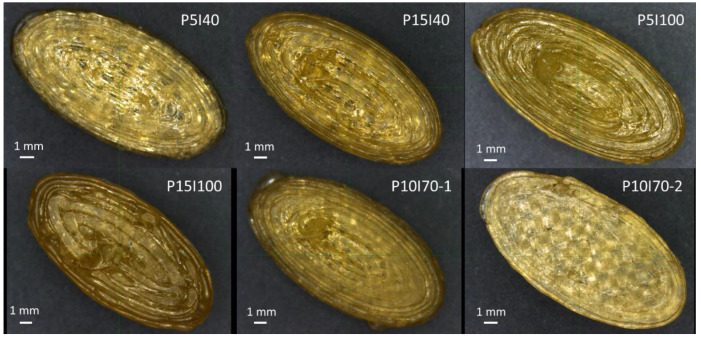
Light microscope images of the tablets from experiments P5I40, P15I40, P5I100, P15I100, P10I70-1, and P10I70-2.

The light microscope images of the tablets from all formulations are presented in Figure 2. The rectilinear infill pattern is most visible for the printlets with 40% infill, followed by those with 70% infill. The image of P10I70-2 represents the side that was adhered to the building plate of the printer in order to demonstrate the difference of the first layer, which was applied with 150% width.

**Figure 3:**
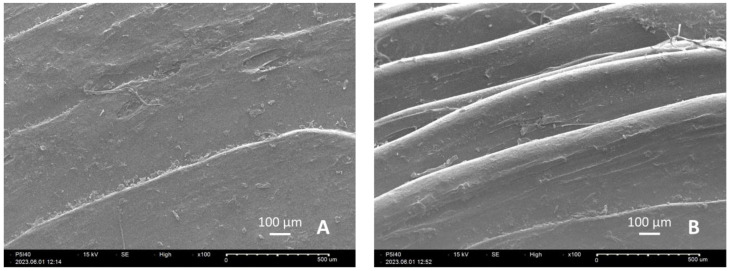
Scanning electron microscope (SEM) image of the surface of tablet P5I40: (**A**) top surface; (**B**) edge surface.

Figure 3 shows the top surface (A) and the edge (B) of a P5I40 tablet. Both images were captured at 100× magnification, which allows observation of the distinctive polymer layers deposited by the printer nozzle. In Figure 3A, it is visible that the top layer is printed with a concentric pattern, as opposed to the rectilinear pattern inside the object. As the tablet is oval-shaped to enhance swallability, the overlapping printing layers of the rounded edge can be observed in Figure 3B.

### 3.3. X-ray Powder Diffraction (XRPD)

**Figure 4:**
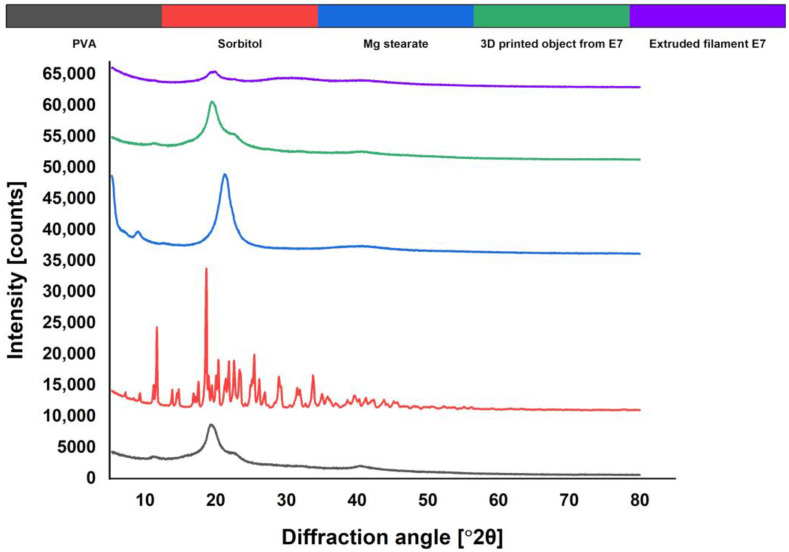
XRPD patterns.

The XRPD patterns of the excipients, the crushed filaments, and the printed discs from a successful formulation (E7) were analyzed and compared. E7 was chosen in order to obtain information about all the excipients that were varied in stage 2 (PVA 2, sorbitol, and magnesium stearate). The diffractograms obtained by XRPD analysis are presented in Figure 4. The XRPD pattern of pure PVA showed a broad peak and a halo of 2θ = 19°–24°, which was in accordance with the literature data and indicated the semi-crystalline nature of the polymer [14]. The pure sorbitol showed two distinct crystalline peaks at 2θ = 11.8° and 18.5°, as well as multiple smaller peaks in the region of 2θ = 20°–34°. The pure magnesium stearate showed a broad peak at 2θ = 21°, but its concentration in the formulation was insufficient to be detected in the diffractograms of the extrudates and the printed discs. The XRPD patterns of the extrudates revealed a broad halo around 2θ = 30°, which indicated that part of the plasticizer was still in a crystalline state. The results for the printed discs did not show a halo in this area and were similar to the patterns of the pure polymer, indicating that the sorbitol was in an amorphous state. The latter confirmed that the plasticizer could be molecularly dispersed in a PVA polymer matrix. Potentially, the presence of crystalline sorbitol in the filaments could be avoided by better mixing during the extrusion process, but this hypothesis needs to be confirmed.

### 3.4. Assay of the Extruded Filaments

The actual drug loading of the extrudates was found to be close to the theoretical: 5.08% for P5, 10.52% for P10, and 14.02 for P15. The slight difference from the expected values of 5%, 10%, and 15% could be explained by the retention of API in the barrel of the extruder [32], but further investigation would be necessary to prove this hypothesis.

### 3.5. Thermogravimetric Analysis

**Figure 5:**
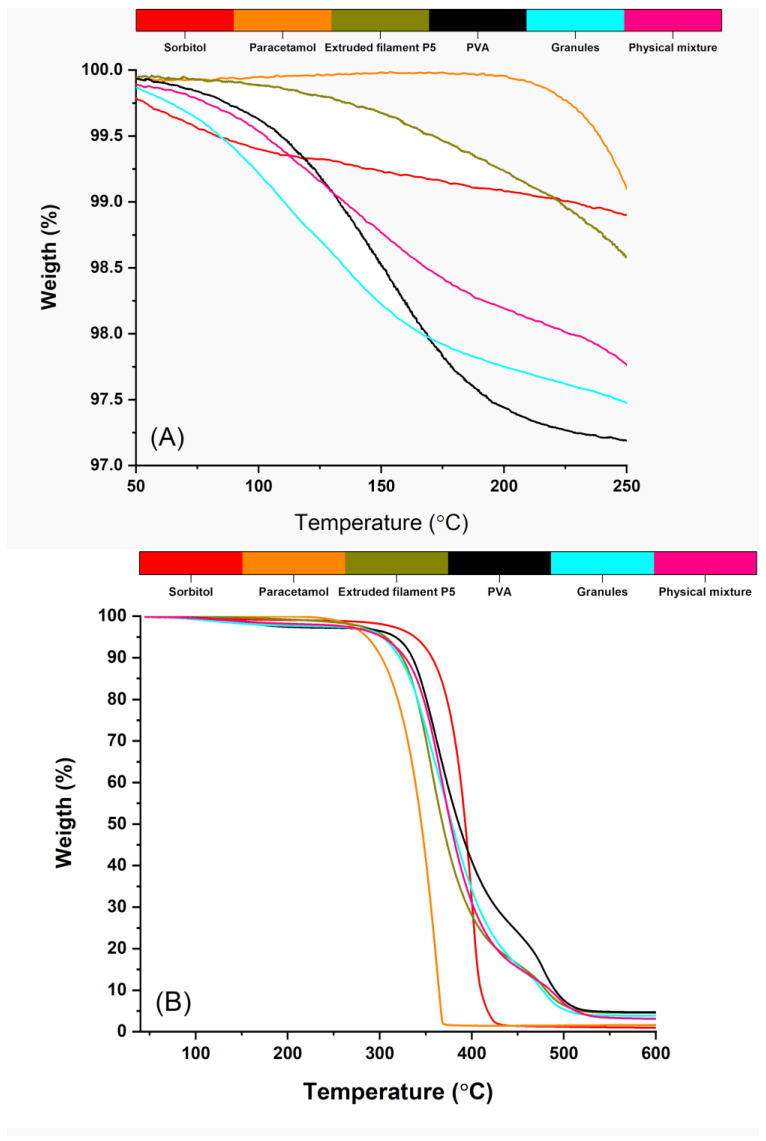
Thermal degradation profiles. (**A**)—results up to 250 °C; (**B**)—results up to 600 °C.

TGA is a suitable method to study both water content and degradation temperature of materials [33]. The thermal analysis of the samples is presented in Figure 5. Up to 250 °C, the observed mass loss was less than 3% (Figure 5A), which could be attributed to the water evaporation. The PVA-containing samples (the pure PVA, the physical mixture, the granules, and the filaments from P5) exhibited more prominent mass loss, which was plausible as the partially hydrolyzed PVA had been reported to contain up to 3.7% water [19]. The mass loss was more significant for the pure PVA, the physical mixture, and the granules (approximately 2.8%, 2.3%, and 2.5%, respectively) and less for the filaments (around 1.5%). This could be explained by the occurring water evaporation caused by the high temperature during the extrusion.

In Figure 5B can be seen that PVA started decomposing around 250 °C, which was observed in other studies [21,32]. Paracetamol showed no degradation until around 230 °C. Sorbitol was thermally stable until 300 °C. Overall, TGA curves for all samples showed that they were stable at the extrusion and the printing temperatures (200 °C and 185 °C, respectively).

### 3.6. Differential Scanning Calorimetry

**Figure 6:**
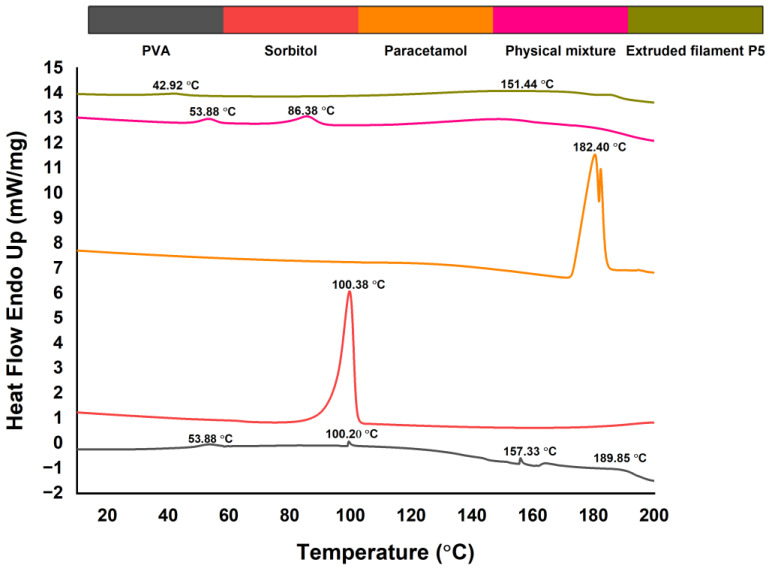
DSC thermograms.

The DSC thermograms are presented in Figure 6. Paracetamol exhibited an endothermal peak at 184.40 °C, indicating the crystalline state of the drug. Sorbitol also showed a distinctive endothermic peak at 100.38 °C. The peak at 53.88 in the PVA endotherm depicted its Tg, while the one at 100.20 °C could be attributed to the water evaporation [19]. Two endothermic peaks indicating the Tm were observed instead of one at 157.33 °C and 189.85 °C. As the PVA used in this study was with a MW in the range between 31,000 and 50,000, these peaks could represent the melting temperatures of the different fractions of the polymer. The broad endotherm also indicated the semi-crystalline nature of the polymer [14], which was confirmed by the XRPD analysis. Expectedly, the physical mixture showed the same endothermic peak at 53.80 °C, indicating that the Tg of the polymer did not change by mere physical mixing with the plasticizer. The second endotherm at 86.38 could potentially be attributed to water evaporation. On the other hand, the extrudates (P5) demonstrated a lower Tg (42.92 °C) and a broader endotherm Tm (151.44 °C), indicating that the melting of PVA and sorbitol during the extrusion resulted in a molecular interaction between both substances. The decrease of Tg proved that in the concentration used, sorbitol had a significant plasticizing effect on the polymer, which was attributed to the formation of hydrogen bonds between the OH-groups of both compounds and a subsequent increase of the distance between the polymer chains [19]. The lower Tg could be the reason for the lower printing temperature (185 °C) compared to the extrusion temperature (200 °C). The absence of the characteristic endotherm peak of paracetamol suggested that the API was included as a solid dispersion within the polymer extrudate.

### 3.7. Rheology

**Figure 7:**
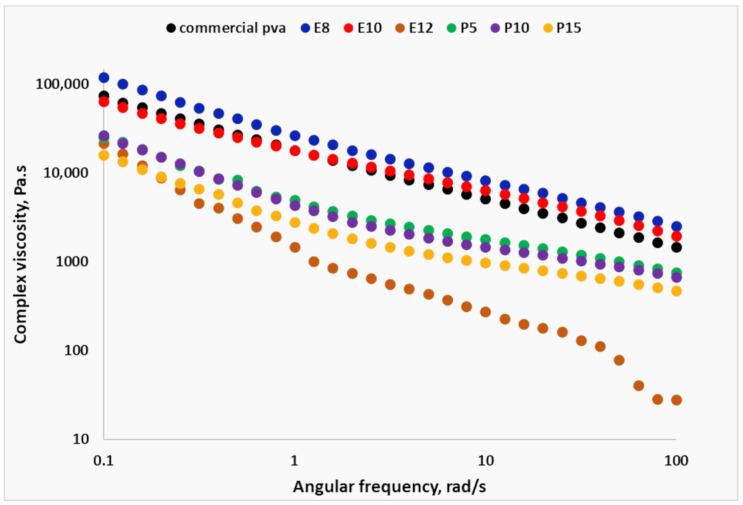
Complex viscosity curves of selected formulations.

In order to assess the printing quality of the selected formulations, their complex viscosity (η*) was compared to that of a commercial PVA filament. The preliminary strain sweep determined that the frequency sweeps should be performed at 0.5% strain, which is within the LVR. Figure 7 shows that in the observed range of angular frequency (ω), the complex viscosity curves do not exhibit a Newtonian plateau, and instead, η* decreases as the frequency increases. The observed shear-thinning was reported to be beneficial for the printing process. It ensured low viscosity within the nozzle, where the material was subjected to high shear rates, and also guaranteed a rapid increase in the polymer viscosity after leaving the nozzle [34]. The formulations E8, E10, and E12 contained approximately 11%, 13%, and 15% sorbitol, respectively. While E10 had a negligibly lower complex viscosity than E8, E12 had a distinctively more pronounced shear-thinning, indicating that adding plasticizer above 13% led to an abrupt change in the properties of the polymer melt. This is in agreement with the empirical observation that E12 was overplasticized. On the other hand, the trend of the complex viscosity curves of E8 and particularly E10 was very similar to the commercial PVA filament, which supported the selection of E10 as an optimal placebo formulation. The addition of paracetamol resulted in a decrease in the complex viscosity compared to the placebo (E10), but they showed the same gradual shear-thinning as E8, E10, and the commercial filament.

### 3.8. In Vitro Drug Release

**Figure 8:**
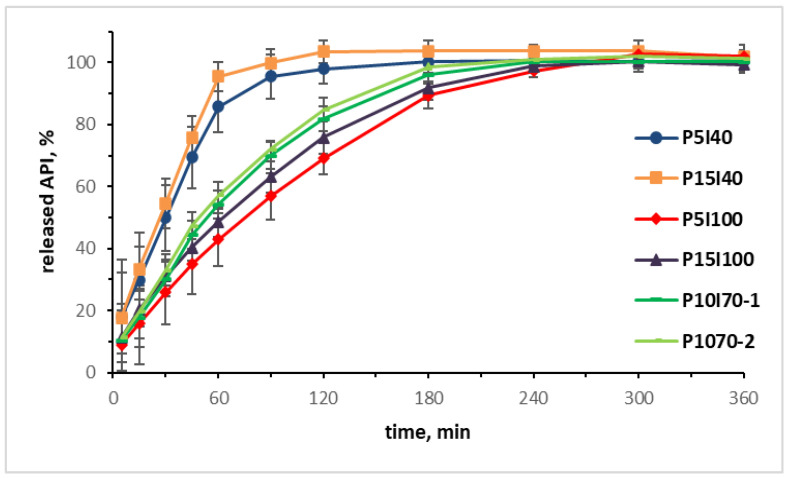
Dissolution profiles of 3D-printed tablets, n = 3 ± SD.

The dissolution profiles obtained from the in vitro release study are presented in Figure 8. Only extrudate P15I40 complied with the requirement that at least 75% of the active substance should be dissolved within 45 min [35]. The results showed that the 3D-printed tablets with higher infill percentages were characterized by a slower drug release. Both formulations with 40% infill generally exhibited a typical immediate-release profile. The tablets with 100% infill were more suitable for a prolonged release, as P5I100 released less than 75% paracetamol after 120 min. This could be explained by the fact that in tablets with lower infill larger surface of the polymer matrix was in contact with the dissolution media and, as also pointed out in previous studies [14,36], the drug release of 3D-printed PVA matrix tablets was defined by a surface erosion mechanism.

### 3.9. Drug Release Kinetics

The release data were fitted to different mathematical models in order to determine the release kinetics. The correlation coefficient (R^2^) was used to compare the different models, where a value closer to 1 indicated a better correlation. In Table 3 are listed the correlation coefficients (R^2^), the constants of the different models, and the release exponent (n) derived after fitting the experimental data of paracetamol release from the samples. From the results presented, it is obvious that paracetamol release from the tablets was a diffusion-controlled process due to the fact that the drug release from the samples was best described by the Higuchi model (R^2^ > 0.98). Also, according to the Higuchi model, the values of k_H_ for samples P5I40 and P15I40 were relatively high (k_H_ > 10), which indicated a significant burst release. This was also proved by drug release studies.

In order to establish the type of the diffusion process, the release data were fitted using the equation derived by Korsmeyer and Peppas and evaluating the value of the release exponent (n). When n ≤ 0.5, the release is based on the Fickian diffusion mechanism; in the case when 0.5 ≤ n ≤1, the release follows the mechanism of an abnormal diffusion (non-Fickian diffusion), and when n > 1, the release follows a complex transport mechanism (super-case-II transport). From the results presented in Table 3, it is obvious that the release followed the mechanism of Fickian diffusion (n ≥ 0.5).

### 3.10. Statistical Evaluation

**Figure 9:**
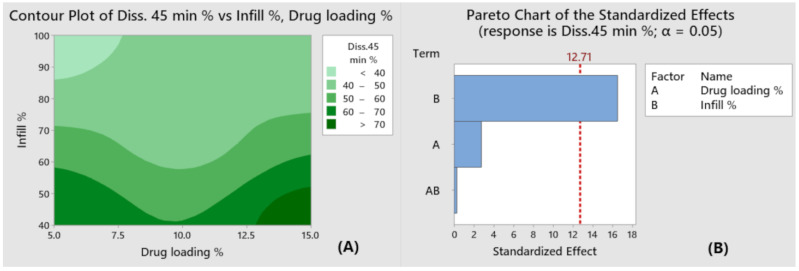
Contour plot (**A**) and Pareto chart of the standardized (**B**) depicting the effect of the independent variables on dissolution rate at 45 min.

The dissolution rate of the tablets with identical infill and higher drug release seemed slightly faster, which indicated that the tablet infill had a significant impact on the drug release. This was visualized by the Contour Plot in Figure 9A with the regions of maxima and minima marked in light green and dark green, respectively.

The two input variables in this study were the drug loading of the extrudates and the infill percentage of the tablets, while the percentage of the released drug after 45 min was set as a response variable. As depicted in the Pareto chart in Figure 9B, only the infill bar crossed the dotted vertical line (positioned at α = 0.05), which indicated that this variable had a significant standardized effect at a 95% confidence level. Analysis of variance (ANOVA) also confirmed that only the infill had a significant effect (*p* < 0.05) on the drug release. The determination coefficient R^2^ was found to be 0.9967, which implied a good prediction accuracy of the given model.

The limitations of our study were the limited capacity of the single screw filament extruders, making it difficult to ensure the homogeneity of the extrudate. A surface roughness phenomenon observed for the PEG-containing formulations should be investigated in more detail to determine whether it was due to the properties of the polyethylene glycol, limitations in the design of the extruder, or whether there was an interaction and superimposition of the effects of other significant factors. Additional research needs to be done for the presence of crystalline sorbitol in the extruded filaments. In addition, a more detailed study concerning the actual drug loading of the extrudates and the possible reasons for the slight difference from the expected values is needed in the future. The printing speed is also a limiting factor, due to which 3D printing is currently considered mainly as a method for the preparation of extemporaneous dosage forms rather than for full-scale manufacturing. Printing with a higher speed and “lower resolution” (greater layer height) or with a printer equipped with multiple nozzles could partially address this limitation.

## 4. Conclusions

Three different partially hydrolyzed grades of PVA were tested as potential excipients for FDM pharmaceutical dosage forms. It was found that PVA with a molecular weight of 31,000–50,000 could be successfully extruded into filaments. Sorbitol, in a concentration between 11% and 13%, was proven as a suitable plasticizer. The presence of a lubricant was not found crucial for the extrusion process. The XRPD analysis established that a fraction of the sorbitol remained in a crystalline state in the extrudates but was in an amorphous state in the printed object. The thermal analysis proved that all compounds were stable at process temperatures and that sorbitol was an efficient plasticizer that reduced Tg and facilitated the extrusion and printing process. Also, the absence of the characteristic endotherm peak of paracetamol suggested that the API was included as a solid dispersion within the polymer extrudate. The optimal placebo and the paracetamol-containing formulations demonstrated complex viscosity curves similar to a commercial PVA filament, which was suggested as an indication of printability. The in vitro drug release study revealed that the formulations with 40% infill generally exhibited typical immediate release profiles, while the tablets with 100% infill were more suitable for a prolonged release regardless of the filament drug loading. The study of the release kinetics established that the paracetamol release from the tablets was a diffusion-controlled process. This study presents an evaluation of the influence of two input variables (the drug loading of the extrudates and the infill percentage of the tablets) on the dissolution rate of the 3D printlets using statistical approaches. The percentage of the released drug after 45 min was set as a response variable. The statistical analysis outlined the tablet infill as the input variable with a statistically significant impact on the dissolution rate (*p* < 0.05).

## Figures and Tables

**Table 1:** Ratios of the excipients in the placebo filaments—stages 1 and 2.

Placebo Filament	PVA 1	PVA 2	PVA 3	PEG	Sorb	MgSt	Comment	Printable
Stage 1								
E1	10	-	-	1.11	-	-	Rough surface Uneven diameter Too brittle to load into printer gear	No
E2	10	-	-	-	1.11	-	Rough surface Uneven diameter Too brittle to load into printer gear	No
E3	-	10	-	1.11	-	-	Rough surface Uneven diameter Can be loaded into printer gear	No
**E4**	**-**	**10**	**-**	**-**	**1.11**	**-**	Smooth surface **Even diameter** **Can be loaded into printer gear**	**Yes**
E5	-	-	10	1.11	-	-	Rough surface Uneven diameter Nozzle blockage	No
E6	-	-	10	-	1.11	-	Smooth surface Even diameter Nozzle blockage	No
Stage 2								
E7	-	10	-	-	1.25	0.15	Smooth surface Even diameter	Yes
E8	-	10	-	-	1.25	-	Smooth surface Even diameter	Yes
E9	-	10	-	-	1.5	0.15	Smooth surface Even diameter	Yes
**E10**	**-**	**10**	**-**	**-**	**1.5**	**-**	**Smooth surface** **Even diameter**	**Yes**
E11	-	10	-	-	1.75	0.15	Rough surface Uneven diameter	No
E12	-	10	-	-	1.75	-	Rough surface Uneven diameter	No

**Table 2:** Design layout with factors and response.

Experimental Run	Factors		Response
	Drug Loading, %	Infill, %	Diss 45 min, %
P5I40	5	40	69.36
P15I40	15	40	75.81
P5I100	5	100	35.00
P15I100	15	100	40.24
P10I70-1	10	70	44.36
P10I70-2	10	70	47.36

**Table 3:** Parameters derived from the different mathematical models describing the drug-release kinetics.

Kinetic Model Printed Tablets	Zero-Order	First-Order	Higuchi	Korsmeyer-Peppas
P5I40	k_0_ = 0.815 R^2^ = 0.930	k_1_ = 0.03 R^2^ = 0.994	k_H_ = 10.708 R^2^ = 0.985	n = 0.064 R^2^ = 0.989
P15I40	k_0_ = 0.839 R^2^ = 0.907	k_1_ = 0.082 R^2^ = 0.978	k_H_ = 10.411 R^2^ = 0.985	n = 0.024 R^2^ = 0.984
P5I100	k_0_ = 0.339 R^2^ = 0.955	k_1_ = 0.014 R^2^ = 0.981	k_H_ = 6.464 R^2^ = 0.992	n = 0.024 R^2^ = 0.996
P15I100	k_0_ = 0.329 R^2^ = 0.935	k_1_ = 0.026 R^2^ = 0.929	k_H_ = 6.416 R^2^ = 0.991	n = 0.013 R^2^ =0.995
P10I70-1	k_0_ = 0.337 R^2^ = 0.909	k_1_ = 0.027 R^2^ = 0.949	k_H_ = 6.594 R^2^ = 0.980	n = 0.022 R^2^ = 0.986
P10I70-2	k_0_ = 0.329 R^2^ = 0.897	k_1_ = 0.028 R^2^ = 0.964	k_H_ = 6.567 R^2^ = 0.986	n = 0.021 R^2^ = 0.985

## Data Availability

Not applicable.
